# Optimization of Glass Transition Temperature and Pot Life of Epoxy Blends Using Response Surface Methodology (RSM)

**DOI:** 10.3390/polym13193304

**Published:** 2021-09-27

**Authors:** Ramli Junid, Januar Parlaungan Siregar, Nor Azam Endot, Jeefferie Abd Razak, Arthur N. Wilkinson

**Affiliations:** 1 College of Engineering, Universiti Malaysia Pahang, Gambang, Kuantan 26300, Pahang, Malaysia; januar@ump.edu.my; 2 Department of Chemistry, Faculty of Science, Universiti Putra Malaysia, Serdang 43400, Selangor, Malaysia; 3 Fakulti Kejuruteraan Pembuatan, Universiti Teknikal Malaysia Melaka, Hang Tuah Jaya, Durian Tunggal 76100, Melaka, Malaysia; jeefferie@utem.edu.my; 4 School of Materials, The University of Manchester, Grosvenor Street, Manchester M13 9PL, UK; arthur.wilkinson@manchester.ac.uk

**Keywords:** epoxy blends, response surface methodology, polymer, crosslinking, pot life, glass transition temperature, central composite design, optimization

## Abstract

The aim of this work was to improve the processability of triglycidyl-*p*-aminophenol (TGPAP) epoxy resin. To achieve this improvement, a diluent, the diglycidyl ether of bisphenol F (DGEBF or BPF), was added to TGPAP, and the blended epoxy was then cured with 4, 4′-diaminodiphenyl sulfones (DDS). A response surface methodology (RSM) was used, with the target response being to achieve a blended resin with a high glass transition temperature (T_g_) and maximum pot life (or processing window, PW). Characterization through dynamic mechanical thermal analysis (DMTA) and using a rheometer indicated that the optimum formulation was obtained at 55.6 wt.% of BPF and a stoichiometric ratio of 0.60. Both values were predicted to give T_g_ at 180 °C and a processing window of up to 136.1 min. The predicted values were verified, with the obtained T_g_ and processing window (PW) being 181.2 ± 0.8 °C and 140 min, respectively, which is close to the values predicted using the RSM.

## 1. Introduction

In a polymer matrix composite, thermosetting polymer resins (e.g., epoxy) are commonly used as matrices to bind reinforcements, as well as to transfer loads during service [1]. These thermosetting polymers exhibit excellent chemical and corrosion resistance, as well as good mechanical strength and thermal properties [2], and consequently are used in many diverse applications including in the automotive industries, electronics (printed circuit boards and semiconductor encapsulants), and as adhesives and composite matrices in aerospace industries [3,4]. Typically, in the aerospace industry, multifunctional epoxy resins like triglycidyl-*p*-aminophenol (TGPAP) are used because they possess a high glass transition temperature (T_g_), due to their ability to crosslink at higher density. Despite showing good properties, this type of liquid epoxy resin has, however, high viscosity, which makes the liquid processing (e.g., for composite fabrication) difficult. As a consequence, blending this type of epoxy resin with another diluent is seen as an option to ease the processing and handling, for example during composite manufacturing [5].

**Figure 1:**
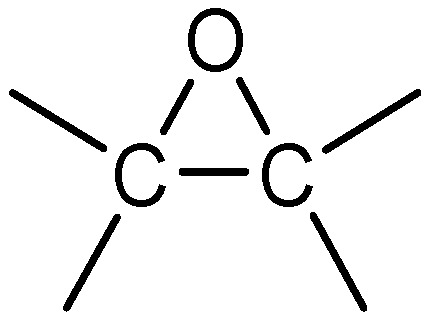
Epoxy functional group.

Epoxy resins are characterized by the epoxy functional group, a three-membered ring (Figure 1) [6]. This is typically derived from epichlorohydrin (a highly reactive compound), and these are termed as glycidyl-based resins [7]. Diglycidyl ether of bisphenol A (BPA or DGEBA) is the most commonly used epoxy resin of this type [8,9]. It is categorized as a bifunctional epoxy resin, in that it has two functional epoxy groups attached to its molecular structure. Diglycidyl ether of bisphenol F (BPF or DGEBF) is another example of a bifunctional epoxy based on glycidyl resin. However, its molecular structure is more flexible and possesses a lower molecular weight, which makes the viscosity lower than the BPA (DGEBA) [10]. This means the epoxy resins based on bisphenol F are normally used as a diluent to reduce the viscosity of other epoxy resins, especially in systems containing epoxy resins of higher functionality (i.e., >2) [7]. As a diluent, DGEBF can aid in processing, for instance allowing for faster degassing (bubble release) with a lower resin viscosity [11,12].

**Figure 2:**
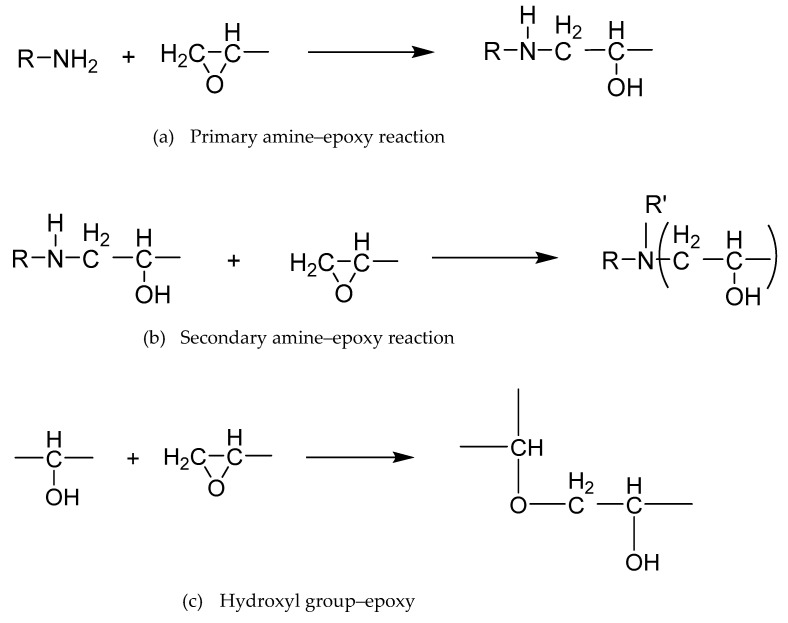
Amine–epoxy reactions (**a**,**b**) and hydroxyl–epoxy reaction (**c**).

Epoxy resins cure by crosslinking via chemical reaction to form three dimensional networks. In order to crosslink, epoxy resins typically need to react with a curing agent (or hardener), most commonly a diamine [7]. Figure 2 shows the reaction scheme between the epoxy groups of the resin and the amine groups of the hardener. The main chemical reactions in Figure 2a,b take place whereby, first, a primary amine reacts with an epoxy to form a secondary amine and a hydroxyl group. After that, further non-linear (branching) reactions can occur, with the secondary amine reacting with an epoxy group [13] to form a tertiary amine. The reaction occurs through the opening of the oxirane ring to form a longer and linear C-O bond [7]. R and R’ in Figure 2b are alkyl groups, where both need not be identical [14]. The hydroxyl group produced may also react with the oxirane ring of the epoxy in a branching reaction, as shown in Figure 2c.

Typically, the formulation of an epoxy blend is studied by the random selection of ratios between an epoxy and the diluent [15]. In some other studies, this ratio was varied using the classical method or named as one-factor-at-a-time, where each factor is varied one at a time, while other factors are held constant [10,16]. The effects of this ratio are then characterized and reported with a graph presentation to show the trend of variation. Even though this approach seems easy to plan and implement, it is, however, not effective. The major disadvantage of this approach is that it fails to show the interactions between factors [17]. In addition, this technique will apply numerous experiments, which are time consuming and may lead to a waste of materials.

A better approach in dealing with several factors (or variables) is running the experimental work through a factorial experiment. This approach is in contrast with the classical method, in that factors are varied together, instead of one at a time. Response surface methodology (RSM) is a design of experiment (DoE) approach and is a useful method that uses mathematical and statistical techniques in which a response is affected by factors (or variables). Through RSM, the interaction between factors can be analyzed and an optimization of response can be determined [18]. The relationship between response and factors can be expressed as

(1) $$y=f\left(x_{1},\;x_{2}\right)+e$$

where, *x*_1_ and *x*_2_ are independent factors, while *y* is the response that depends on both factors. The above relation can be read as the dependent factor *y* being a function of *x*_1_, *x*_2_, and experimental error, denoted as *e*. Error, *e*, represents the noise or any measurement error not counted in *f*. If the response is correlated with variables using a linear function, the function is a first-order model [19,20], sometimes also known as a main effects model. For the case of two independent factors, a first-order model can be written as

(2) $$y=\beta_{0}+\beta_{1}x_{1}+\beta_{2}x_{2}+e$$

If between these two factors, an interaction exists, that interaction can be added to the function. This will be

(3) $$y=\beta_{0}+\beta_{1}x_{1}+\beta_{2}x_{2}+\beta_{12}x_{1}x_{2}+e$$

If the response surface produces a curvature, a higher degree of polynomial is used. This function is known as a second-order model. For two independent factors, the function can be expressed as

(4) $$y=\beta_{0}+\beta_{1}x_{1}+\beta_{2}x_{2}+\beta_{11}x_{1^{2}}+\beta_{22}x_{2^{2}}+\beta_{12}x_{1}x_{2}+e$$

Previously, Guo et al. [21] added the reactive diluent 692 (Benzyl Glycidyl Ether) into epoxy resin to study its influence on workability and the effect on the mechanical properties of the host material. They reported a 15% increase in liquid flow-ability when the diluent was added to the system, which enhanced the processing capability. Mechanically, they also reported that properties including the toughness and compressive strength of the blends all increased significantly. In another study, Harani et al. [22] added hydroxyl-terminated polyester resin (polyols) at different concentrations into epoxy resin, with the aim of reducing the viscosity and increasing the mechanical properties. Positively, their findings showed an increase in impact properties, lower viscosity, and longer pot life of the blended mixtures. They reasoned that the improvement in impact strength with the addition of polyols to the epoxy was due to the increase in the degree of entanglement within the mixture, which improved the strength against failures. On the other hand, a lower viscosity was exhibited by the liquid after blending, as a typical behavior when a liquid with a higher viscosity is added to a liquid with lower viscosity, producing a blended liquid with a viscosity between the two.

Herein, we aim to demonstrate the workability of TGPAP-BPF-DDS amine mixtures and how these effects are simplified by incorporating the response surface methodology (RSM) approach as the DoE. The requirements of this work were: (1) to find an epoxy blend at high T_g_ with minimum viscosity, and (2) to achieve the maximum pot life (or processing window, PW) of the epoxy blend. Despite there being a few papers reporting work related to epoxy blending, to the best of our knowledge, pot life was rarely discussed, and optimization using a design of experiment (DoE) approach for the blending of epoxy resin has also not been reported to date. The blended epoxy resin in this work is expected to offer the desired performance once cured and facilitate composite processing in later work, where it will be presented in a forthcoming paper.

## 2. Materials and Methods

### 2.1. Epoxy Resin

**Figure 3:**
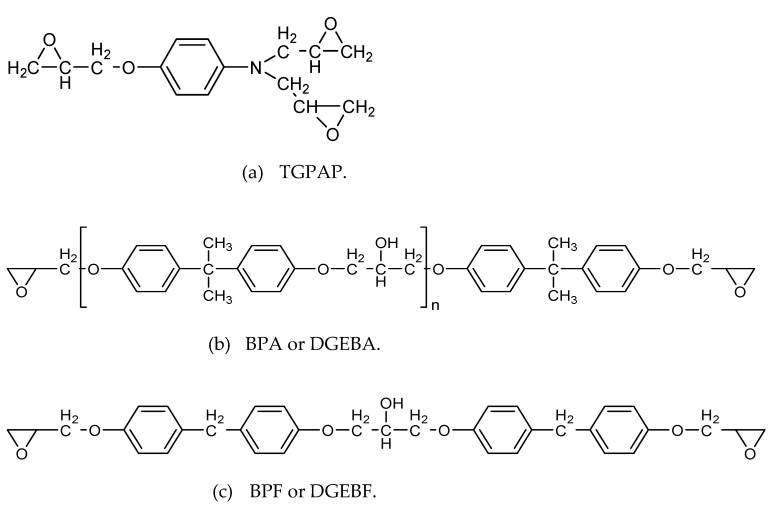
Chemical structure of (**a**) TGPAP, (**b**) BPA or DGEBAand (**c**) BPF or DGEBF.

The epoxy resins used in this study were triglycidyl-*p*-aminophenol (TGPAP) and diglycidyl ether of bisphenol F (BPF). In this work, BPF is also denoted as DGEBF. The chemical structures for both resins are shown in Figure 3. General information on the epoxy resins is given in Table 1.

### 2.2. Hardener

**Figure 4:**
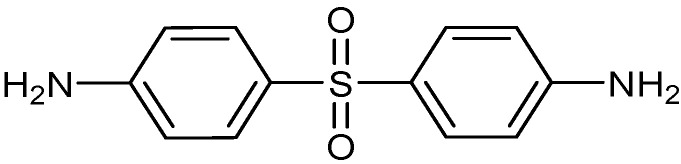
Chemical structure of 4, 4′-diaminodiphenyl sulfone (DDS).

The type of hardener used to cure the blended epoxies in this work was 4,4′-diaminodiphenyl sulfone (DDS). DDS exists in the form of a white powder and is suitable for aerospace engineering applications. The chemical structure of this hardener is shown in Figure 4 [7]. The properties of this hardener are indicated in Table 1.

### 2.3. Resin Mixtures

**Figure 5:**
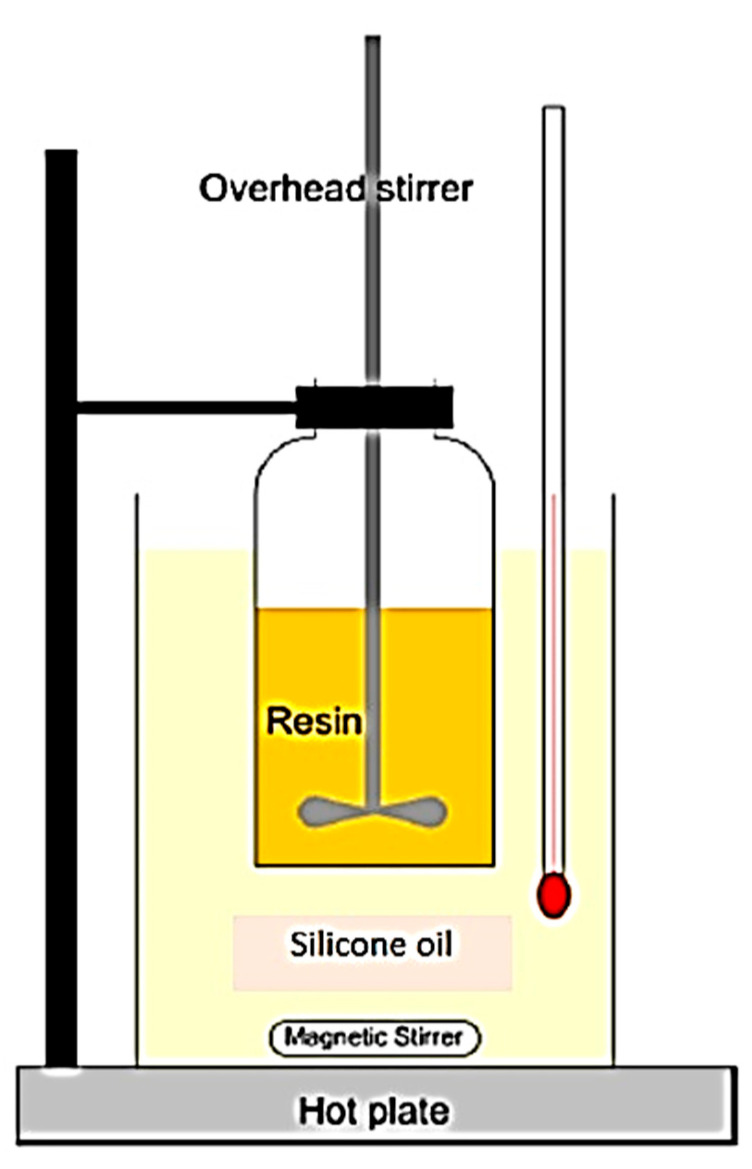
Epoxy blending setup.

Blending of epoxy resin was carried out using a mechanical stirrer at 2000 rpm for 30 min in a silicone oil bath, which was heated to around 100 °C. This setup is drawn schematically in Figure 5. This process produced a miscible transparent mixture with a light-yellow color. The blend consisted of TGPAP epoxy, BPF as a diluent, and DDS as hardener. The amount of diluent (BPF) was obtained from subtraction of 100% TGPAP. The quantities of hardeners were determined from stoichiometry calculations using Equations (5)–(7), where [ ] refers to concentration, EEW is epoxy equivalent weight, wt_TGPAP_ is the weight percentage of TGPAP, and wt_BPF_ is the weight percentage of BPF (or DGEBF) [23].

(5) $$\mathrm{Stoichiometry},\;X2=\frac{\left[\mathrm{Amine}\right]}{\left[\mathrm{Epoxy}\right]}$$

(6) $$\left[\mathrm{Epoxy}\right]=\frac{{\mathrm{wt}}_{\mathrm{TGPAP}}}{{\mathrm{EEW}}_{\mathrm{TGPAP}}}+\frac{{\mathrm{wt}}_{\mathrm{BPF}}}{{\mathrm{EEW}}_{\mathrm{BPF}}}$$

(7) $$\left[\mathrm{amine}\right]=\;\mathrm{stoichiometry}\;\times \left[\;\frac{{\mathrm{wt}}_{\mathrm{TGPAP}}}{{\mathrm{EEW}}_{\mathrm{TGPAP}}}+\frac{{\mathrm{wt}}_{\mathrm{BPF}}}{{\mathrm{EEW}}_{\mathrm{BPF}}}\right]$$

### 2.4. Design of Experiment

In this work, two factors were studied. The first was the BPF content (wt.%) in the epoxy blend (factor A). The second (factor B) was the stoichiometric ratio (g/g) between the amine hardener and epoxy. A DoE approach was carried out using a RSM assisted by Design Expert^®^ software version 7.1. An efficient design tool, known as central composite design (CCD), was chosen for this study.

**Figure 6:**
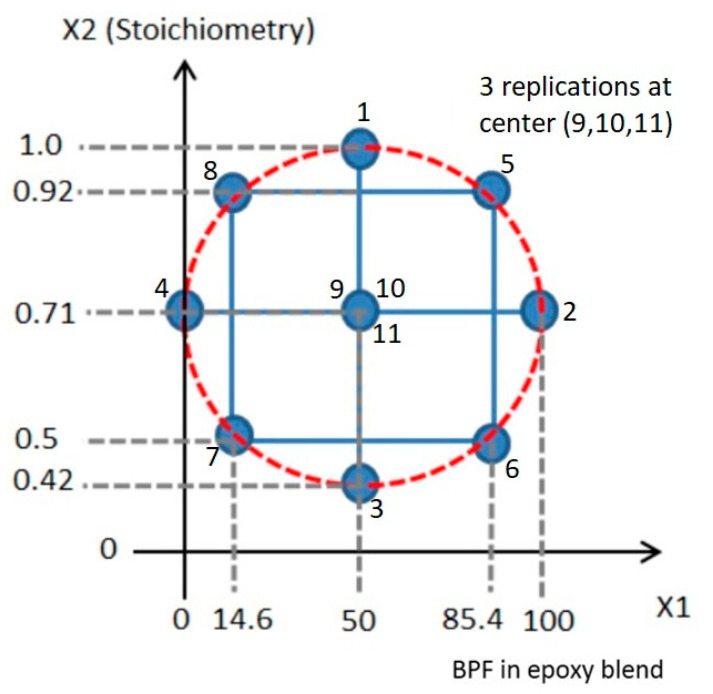
Actual values in the CCD.

In this work, the number of variables (or factors), *k*, was 2, namely the BPF content in the epoxy blend (A) and the stoichiometric ratio of amine to epoxy groups (B). The CCD used had a core two-level factorial design, in which one level is referred to as a low level (lower bound of the actual value, coded as −1) and the other as the high level (upper bound of the actual value, coded as +1) of each variable. The number of experiments required for this CCD was calculated as 2*^k^* for the two-level factorial cube points, 2*^k^* for the axial or star points (which extend the measured experimental volume beyond that of the two-level factorial), and several replication samples at the center of the two-level experimental volume, to allow estimation of the curvature of standard error. It is recommended to have 3 or 5 replicates at the center of the experimental region [19]. In this work, 3 were used. Hence, the total number of experiments was 2^2^ + 2^2^ + 3 = 11. Figure 6 shows the construction of the CCD design with actual values, which consists of 4 axial points (1, 2, 3, and 4, as in Figure 6), 4 cube points (5, 6, 7, and 8), and 3 replicate points (9, 10, and 11) at the center. These values are presented in Table 2 as factor 1 (A) and factor 2 (B). The coded values to represent each actual value are −1 (low level), +1 (high level), and 0 (middle level).

**Figure 7:**
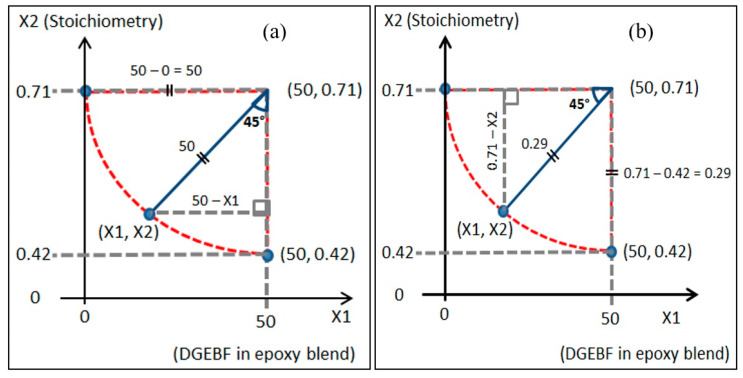
Cube point (−1, −1) to determine the actual value for (**a**) X1, and (**b**) X2.

Each point for an actual value, as indicated in Figure 6, was determined according to the location of that point. Axial points (1, 2, 3, and 4) were determined based on the minimum and maximum value of the range of the factor; for example, in factor 1 (A), 0% is the minimum value (low level, −1) and 100% is the maximum value (high level, +1) of BPF content in BPF/TGPAP content. Cube points (5, 6, 7, and 8) were determined using trigonometric calculation. Figure 7 shows an example of the calculation to determine the actual value of the cube point at point 7.


From Figure 7a, X1:
From Figure 7b, X2:


$\sin45{}^{\circ}=\frac{50-X1}{50}$



$\sin45{}^{\circ}=\frac{0.71-X2}{0.29}$



50−X1=50 sin45



0.71−X2=0.29 sin45



50−X1=35.4



0.71−X2=0.21



X1=50−35.4



X2=0.71−0.21



X1=14.6



X2=0.50




From the trigonometry calculation, the actual value for the cube point at point 7 with a coded value (−1, −1) was (14.6, 0.50), and the values for the other 3 cube points (point 5, 6, and 8) were determined using the same calculation. The actual values for the 3 replication points (9, 10, and 11) with a coded point (0, 0) in CCD were determined as the middle value in a range from minimum to maximum of the factor in actual values. For example, the actual value for the replication points for factor 1 (A) is 50%, when the range is 0% to 100% BPF of BPF/TGPAP content. These 11 values are listed in Table 2 as Factor 1 (A) and Factor 2 (B).

### 2.5. Testing Procedures

The response from the input factors in this work was T_g_ and PW (pot life). For curing, blended epoxy was poured into a flexible mold made from polytetrafluoroethylene (PTFE)-coated fabric and degassed in a vacuum oven to eliminate trapped air at 100 °C for ~30 min. The degassed epoxy was then put into a circulating-air oven and heated following a cure cycle of 2 h at 130 °C, 2 h at 160 °C, and 2 h at 200 °C, followed by a post cure for 5 h at 200 °C. T_g_ was measured using a dynamic mechanical thermal analyzer (DMTA), where the T_g_ was determined from the onset of storage modulus curves. DMTA was performed using a Perkin Elmer 8000 from Perkin Elmer Ltd., Beaconsfield, United Kingdom, with a 3-point bending mode at a frequency of 1 Hz. Rectangular specimens (40 mm × 10 mm × 3 mm) ± 1 mm were cut using a Benetec sliding cutter. Specimens were heated from 30 °C to 300 °C at a rate of 5 °C/min. The curves of E′, E″, and tan δ were displayed as a function of temperature using Pyris software (Perkin Elmer). Three repetitions (n = 3) were performed for each run of experiments, to ensure reproducibility.

The PW of the epoxy blends was defined as the length of time for which the epoxy can retain a viscosity low enough for it to be processed. In this work, the PW was determined using rheometer and defined as the time for the epoxy blend to reach a viscosity of 100 Pa s, as stated in ASTM D 4473-95 [24]. The rheometer used was Haake MARS (modular advanced rheometer system) from Thermo Scientific, Karlsruhe, Germany. Viscosity measurements in oscillatory shear mode were carried out by placing the epoxy between two parallel plates of 35 mm diameter fitted to the upper measuring head and the fixed lower mount. The distance between the plates was then closed to give a 0.5 mm gap, and the furnace was closed around the plates. For curing, the specimen was first heated to 80 °C, at which point measurements began at a 1 Hz oscillation frequency with a controlled stress at 2.0 Pa. The temperature was increased further by 10 °C/min until 130 °C and then remained constant during curing of the specimen. The value of PW was taken from the graph, where the viscosity was 100 Pa s. At least 3 repetitions were carried for every run in the CCD, to ensure reproducibility.

## 3. Results

### 3.1. Glass Transition Temperature

The presented values shown in Table 2 indicate the results for both T_g_ and PW, based on BPF content in the epoxy blend and stoichiometric ratio.

**Figure 8:**
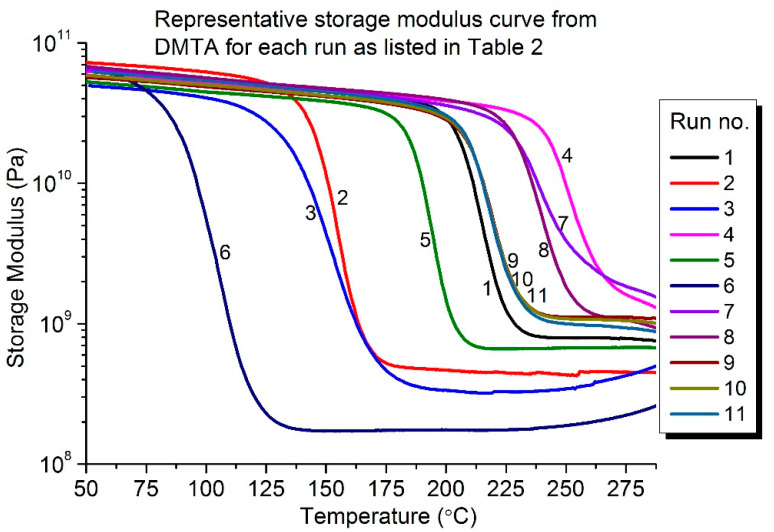
Representative storage modulus curve from DMTA for each run, as listed in Table 2.

**Figure 9:**
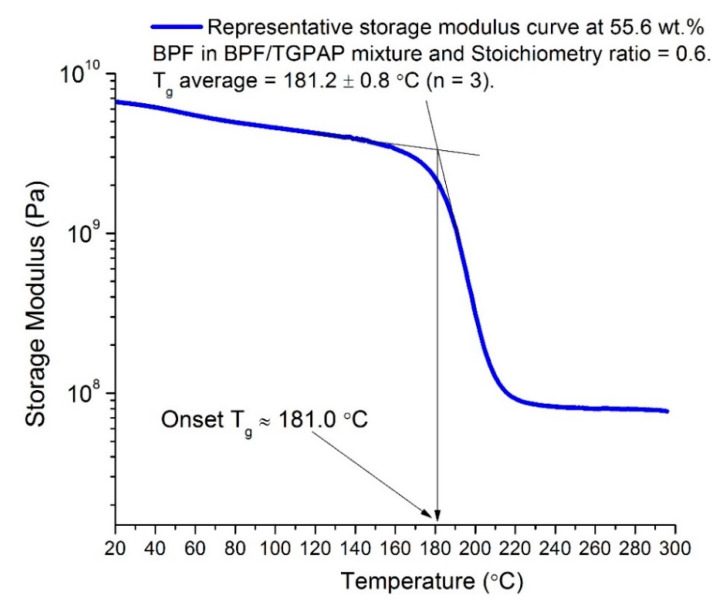
Determination of onset T_g_ from a representative storage modulus curve at 55.6 wt.% BPF epoxy blend and stoichiometry ratio = 0.6.

Figure 8 shows the representative storage modulus curve for each sample for all 11 runs, as listed in Table 2. As mentioned earlier in Section 2.5, T_g_ was determined from the onset of the storage modulus curve. These T_g_ values are recorded in Table 2 as response 1. An example of how the onset T_g_ was determined is presented in Figure 9.

**Figure 10:**
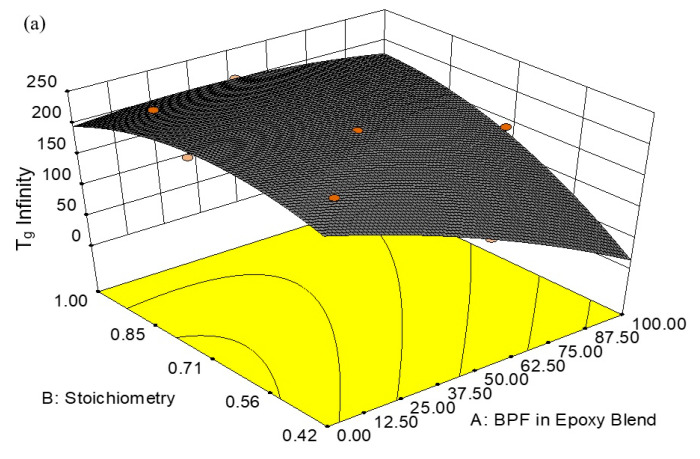

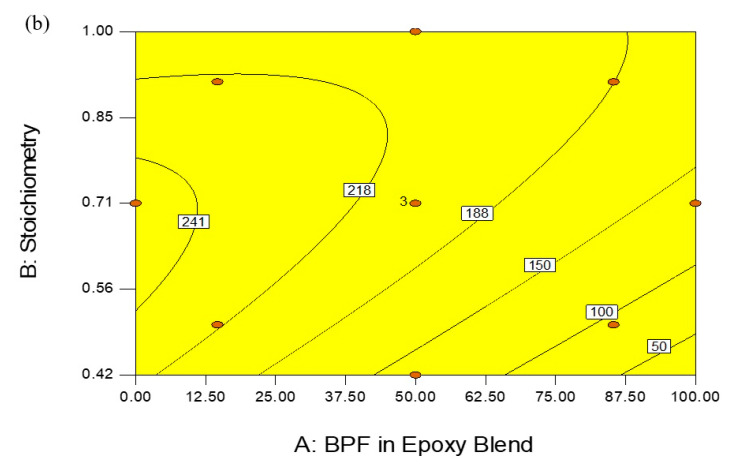
Response surface for T_g_ of BPF/TGPAP/DDS epoxy resins (**a**) 3D plot and (**b**) contour plot.

The effect of BPF content and the stoichiometry ratio on the value of T_g_ is shown in Figure 10. As seen in the 3D and contour plots of surface response, T_g_ increases as the amount of TGPAP epoxy increases in the epoxy blend (factor A). For example, at a fixed stoichiometry ratio of 0.42, T_g_ increased by 336% when the TGPAP increased from 12.5% to 95% in the epoxy blend (Figure 10b). This is due to the fact that TGPAP has a higher functionality compared to a bifunctional epoxy such as BPF, resulting in a greater crosslink density. Therefore, a higher T_g_ was obtained in the epoxy blend with a higher amount of TGPAP.

On the other hand, for a fixed amount of BPF in the epoxy blend, as the stoichiometry increases (factor B), the T_g_ increases. For example, at a fixed amount of BPF = 87.50% in the epoxy blend, the T_g_ increased by 200% when the stoichiometry ratio increased from 0.42 to 0.71 in the epoxy blend (Figure 10b). However, beyond a stoichiometry of 0.71 and at a lower amount of BPF in the epoxy blend, the T_g_ value was observed to decrease. This was probably due to an increased number of secondary amines, due to their lower reactivity and possible steric hindrance possibly not fully reacting with the epoxide groups [25]. Thus, an increased amine content may reduce the value of T_g_ in an epoxy system by reducing the crosslink density and increasing the free volume [26].

This finding is in agreement with the study by Palmese and Mccullough [27], who reported a significant effect on the modulus and T_g_ with a variation in stoichiometry ratio (r). They studied the effect of varying the amount of hardener added to DGEBA and showed that the highest T_g_ was obtained at 30 parts per hundred (pph), beyond which the T_g_ reduced. They mentioned that using the wrong stoichiometry ratio of curing agent (either more or less) caused the final structure of cured composites to have a lower crosslinking density, therefore lowering the T_g_. Guerrero et al. [28] also reported a similar pattern of T_g_ for epoxy resin cured at a different stoichiometry ratio, r, which ranged from 0.3 to 1.0. They found the maximum T_g_ was obtained when r = 0.8–0.9, and decreased thereafter. The following equations are the final empirical regression models generated by the Design Expert^®^ software for T_g_ in both coded and actual factors. Note that in the equation, BPF is the short term used to represent the weight of BPF in the total weight in the epoxy blend.

(8) $$Coded\;Factor=226.53\;–\;18.61A+10.95B+14.39AB\;–\;2.84A^{2}\;–\;18.66B^{2}$$

(9) $$\begin{array}{cc} Actual\;Factor= & 56.01\;–\;2.85\;*\;BPF+584.26\;*\;Stoichiometry\\ & +3.31\;*\;BPF\;*\;Stoichiometry\;–\;0.0063\;*\;BPF^{2}\;–\;443.72\;*\;Stoichiometry^{2} \end{array}$$

The relative impact of each factor (A and B) to response 1 (T_g_) can be identified by observing each factor coefficient. The coefficient as indicated in Equation (8) for the coded factor represents the expected change in response 1 (T_g_) per unit change of each factor (A and B). For Equation (8) of the coded factor, A and B are the main effects, AB is the two-level interaction effect, and A^2^ and B^2^ are the second order effects. According to the regression model generated using Design Expert^®^ software, the two-level interaction between BPF content in the epoxy blend (A) and stoichiometry (B) is the most significant factor associated with the T_g_. This interaction produces the highest value of coefficient, of +14.39 of the coded value [29]. In addition, note that the contours are curved as the model contains a strong interaction term. In contrast, the equation for the actual factor (Equation (9)) is used to make predictions about the actual value of response 1 (T_g_) for given actual values of each factor (A and B). This means that the value for each factor for Equation (9) should be specified in the actual values. The response (T_g_) and factors (A and B), as indicated in the 3D plot and contour plot in Figure 8, are based on a prediction from the model in Equation (9) for actual factors. Unlike Equation (8), Equation (9) should not be used to compare the relative impact of each factor, since the coefficients in this equation are scaled to accommodate the units of each factor and the intercept is not at the center of the design space [30]. The values shown in the 3D plot (Figure 10a) and contour plot (Figure 10b) exhibited the actual values as modeled by Equation (9) (actual factor).

**Figure 11:**
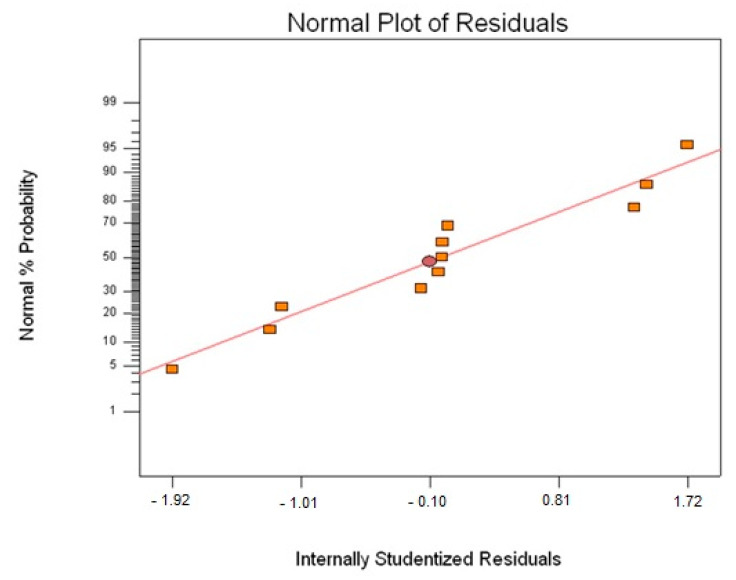
Normal probability plot of residual for T_g_ data (rectangular plots are the 11 points in the CCD).

Figure 11 shows a normal probability plot of the residual in response to the T_g_ value. It is a plot to check whether the data set is normally distributed or not. From the plotted data, the normal distribution of the points should approximately form a straight line [31]. Checking the plots for T_g_ clearly indicates that no outlier residuals are far from the straight line. This is desired and shows that the errors are distributed normally [31].

A summary of the test for T_g_ is presented in Table 3. The confidence level, denoted as P, is usually selected at 95% [32]. The value of “P > F” in Table 3 for the model is less than 0.05 and suggests that the model is significant. Similarly, all other model terms are significant since the “prob > F” for each of these terms is also less than 0.05. Therefore, model reduction is not required.

As mentioned previously, different amounts of TGPAP in the epoxy blend affect the T_g_ of the cured epoxy blend, due to the different functionality. In addition, as was discussed earlier, different amounts of amine hardener being added to the blended epoxy also produce a significant effect on the T_g_ [27]. Both factors A and B have a significant effect on the T_g_, and hence both the two-level interaction (AB) and the second-order effect are also significant. The coefficient of determination R^2^ value was high, close to 1, which is desirable. Adequate precision measures the signal-to-noise ratio, and a ratio greater than four is desirable [33,34]. For this model, the value of adequate precision was 31.541, indicating an adequate signal [31].

### 3.2. Pot Life (Processing Window, PW)

**Figure 12:**
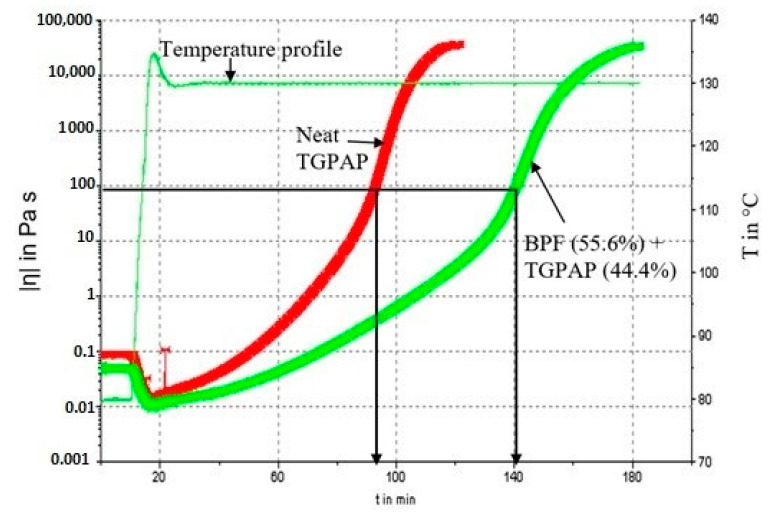
Representative isothermal complex viscosity profile for measuring the PW for curing of neat TGPAP (PW ≈ 97 min) and a 55.6 wt.% BPF in epoxy blend (PW ≈ 140 min).

In this work, the PW was defined as the length of time until the viscosity of an epoxy blend increases and reaches 100 Pa s. Again, Table 2 shows the experimental results for the PW (response 2) and Figure 12 shows an example of the viscosity profiles recorded for two epoxy systems (at the same stoichiometric ratio of 0.60) for measuring the PW. The behavior of the epoxy resin during the measurement can be explained as follows. At the start of the experiment, the temperature of the rheometer was set at 80 °C. The initial viscosity of neat TGPAP epoxy (~0.100 Pa s) was higher than the formulated epoxy blend (~0.080 Pa s), due to the addition of a lower viscosity BPF to the blended epoxy. After 10 min at 80 °C, the temperature of the furnace was raised to 130 °C, and the viscosity of the resin was seen to decrease. This is a normal behavior of a liquid, where higher temperature will cause viscosity to decrease. The minimum viscosity recorded for both profiles was approximately 0.010 Pa s. As the temperature was raised and maintained at 130 °C, the viscosity profile increased due to network formation (crosslinking) and finally reached a plateau, suggesting the system was completely vitrified. In this work, the PW was taken as the time at which the viscosity of the resin reached 100 Pa s, following ASTM D4473–95a, standard test method for plastics: dynamic mechanical properties, cure behavior [24].

**Figure 13:**
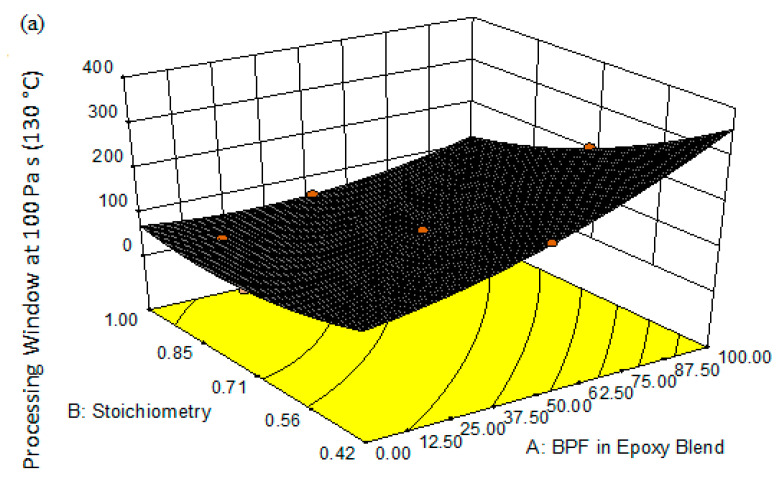

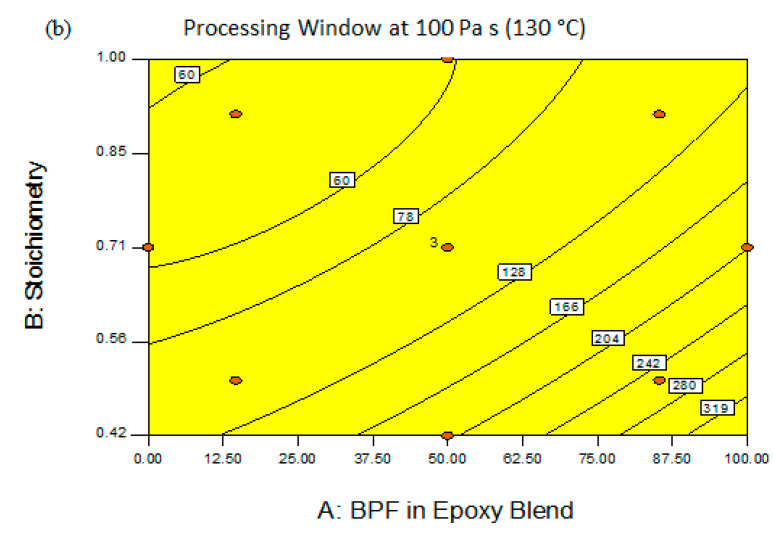
Response surface for PW of BPF/TGPAP/DDS epoxy blend resins (**a**) 3D plot, and (**b**) contour plot.

Figure 13a,b show the 3D plots and contour plot of the response surface of the PW for the interaction between factor A (BPF in epoxy blend) and factor B (stoichiometry ratio). It can be seen from Figure 13 that a higher BPF (less TGPAP) in the epoxy blend resulted in a longer time for the resin to reach 100 Pa s. For example, at a fixed stoichiometry ratio of 0.42, the PW increased by 59% when the BPF increased from 12.5% to 52% in the epoxy blend (Figure 13b). This means more time will be available to process the blended epoxy; for example, during the fabrication and manufacturing of composites. As mentioned previously, TGPAP’s nominal functionality of 3 is higher than that of the bi-functional BPF. Therefore, as expected, a higher amount of TGPAP in the epoxy blend will reduce the PW due to the system reaching the gelation level earlier [35].

Small increases in PW are observed towards the end of the area in the top-left corner of the response plot in Figure 13a,b. The formation of this contour, which shows a small increase in PW in that area, was probably influenced by the value of PW obtained for that point (14.6, 0.92). This result could be due to experimental error, as indicated by the error value (standard deviation), which is anomalous. During experiments, the control temperature of the rheometer furnace was within a range of ±10 °C. The fluctuation in control temperature within this range could possibly affect the rate of crosslinking and influence the value of PW for each test with the same sample, which would finally affect the average value. In addition, to minimize the errors in future, it is recommended that the three repeats of the test for each sample should be performed at the closest possible time to each other. Otherwise, a reaction between epoxy and amine could possibly occur if the epoxy blend was left unused, even if the sample was placed in the freezer. This could possibly result in crosslinking prior to the experiment, which would affect the measured values of the processing window.

The following equations are the final empirical regression models generated by the Design Expert^®^ software for PW in both coded and actual factors:

(10) $$Coded\;Factor=71.08+18.87A-36.03B-14.47AB+6.43A^{2}+17.49B^{2}$$

(11) $$\begin{array}{cc} Actual\;Factor= & 320.84+2.39\;*\;BPF-666.59\;*\;Stoichiometry-3.33\;*\;BPF\;*\;\\ & Stoichiometry+0.0143\;*\;BPF^{\;2\;}+415.99\;*\;Stoichiometry^{\;2\;} \end{array}$$

From the regression model, the most significant factor is the level of BPF in the epoxy blend at a coefficient of +18.87. The same explanation for Equations (8) and (9) of coded and actual factors for response 1 (T_g_) applies here for the explanation of Equations (10) and (11) for response 2 (PW).

**Figure 14:**
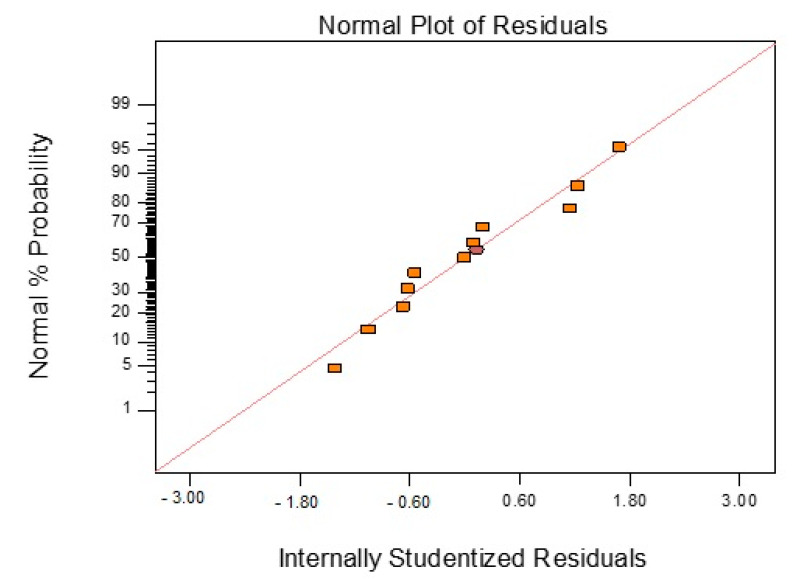
Normal probability plot of residual of PW data (rectangular plots are the point in CCD).

Figure 14 shows the normal probability plot of the residual in response to changes in the PW. As explained previously for the T_g_ response, similarly for the case of PW, it can be observed that all normal plots for PW are scattered around the straight line, which is desirable and illustrates that the distribution of errors is normal [31].

The analysis of variance (ANOVA) for the PW is shown in Table 4. The value of “Prob > F” is less than 0.05, which indicates that the model is significant. The same explanations as in the previous subsection for T_g_ are applied to describe the ANOVA analysis (Table 4).

### 3.3. Optimization of Epoxy Formulation

In order to optimize the system, a few criteria were set as goals [36]. Table 5 shows the criteria used to limit the range of the factors, to achieve the optimum goal for each response. The explanation of each criterion is as follows: the BPF level in the epoxy blend was set to “maximize”, since more BPF in an epoxy blend will give a lower viscosity for a better processability of polymer. The stoichiometry was set to “minimize” to reduce the viscosity of the epoxy blend. A higher stoichiometry will result in the addition of a higher amount of DDS powder, which will raise the viscosity of the epoxy. An increase in viscosity will be a disadvantage for polymer processing. The responses considered were T_g_ and PW. It has been reported that for structural aerospace applications polymer composites should have a T_g_ value of at least 180 °C, due to high temperature applications during service [37]. Therefore, the T_g_ was set to a range between 180 and 185 °C for the optimization. Finally, the PW was set to be “maximize”, as the epoxy resin system needs to be processed before it starts to cure and its viscosity increases significantly. The importance column (as shown in Table 5) and * symbol reflect the priority of achieving optimization [38].

**Figure 15:**
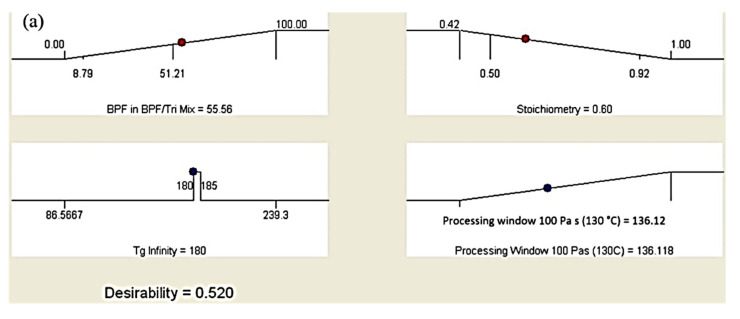

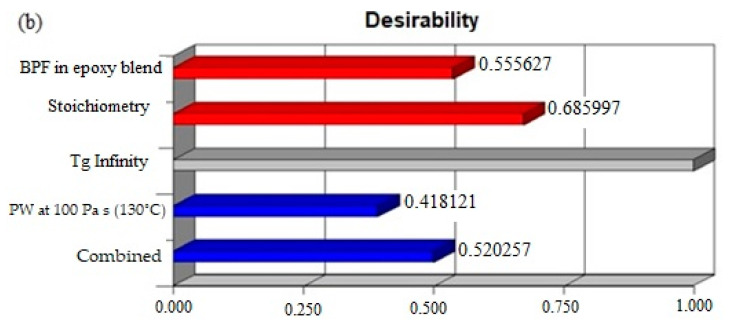
Desirability chart in (**a**) ramp mode of factors for optimum responses of the epoxy blend, and (**b**) histogram chart with factors and predicted responses.

Optimization targeting the maximum goal yielded nine suggested solutions for the epoxy formulation. However, the epoxy formulation with the highest desirability was selected. In Design Expert^®^ software, the desirability chart can be viewed in two modes. First, in a ramp mode, as shown in Figure 15a. The view of the desirability chart in ramp mode will indicate the individual elements for easier interpretation, predicted in actual values. Each ramp in Figure 15a shows a point which reflects the optimum value for factors and responses for that solution. The criteria, which were set as in Table 5, to achieve the optimum response yielded the factor value at 55.6% of BPF in the epoxy blend (factor A) and a stoichiometry of 0.60 (factor B). Both values are predicted to give T_g_ at 180 °C and PW at 136.1 min (Figure 15a). Second, the desirability value can be viewed in histogram chart (Figure 15b). This form of graph indicates how well each variable satisfied the criteria, with values near to 1 being good. The desirability for each factor and responses were also indicated, as in Figure 15b. In this work, the combined desirability was predicted using Design Expert^®^ at a value of 0.520. A verification test was carried out, which gave a T_g_ at 181.2 ± 0.8 °C (as in Figure 9), which is close to the predicted value (180 °C in Figure 15a). For PW, the verification run gave a PW of ≈140 min (as in Figure 12). The discrepancy between the verification test and predicted value (136.1 min as in Figure 15a) for PW could have been associated with the range of the control temperature in the furnace, which was within ±10 °C. As explained previously, a fluctuating temperature within this range could have affected the test result.

## 4. Conclusions

An optimum formulation for a epoxy blend of TGPAP and BPF, with the respective stoichiometry ratio (r), was determined in this work using RSM. The T_g_ and PW of the blended epoxy were studied in this work, with the target response being to achieve a resin with high T_g_ and maximum PW. The result indicates that optimization can be achieved at 55.6 wt.% of BPF and a stoichiometric ratio of 0.60, giving the predicted values of 180 °C for T_g_ and 136.1 min for PW. These values were verified, which produced a T_g_ of 181.2 ± 0.8 °C and ~140 min for PW. The optimized epoxy blends obtained in this work will be carried forward to the next phase in our study, which will be presented in a forthcoming paper.

## Figures and Tables

**Table 1:** Properties of the resins and hardener used in this study.

Materials	Trade Name	Equivalent Weight (g/eq)	Density (g/cm^3^)	Supplier
Triglycidyl p-aminophenol (TGPAP)	Araldite MY0500	101	1.21–1.22	Huntsman
Diglycidyl ether of bisphenol F (BPF)	DER 354	164	1.19	Dow Chemical
4,4′-diaminodiphenyl sulfones	DDS	62.08	1.36	Huntsman

**Table 2:** Experimental results from the experimental design, n = 3. (Value of glass transition temperature was measured from the onset of the storage modulus curve).

Run	Factor 1	Factor 2	Response 1	Response 2
	A: BPF in BPF/TGPAP (wt.%)	B: Stoichiometry Amine:Epoxy (g/g)	Glass Transition Temperature (T_g_)	Processing Window (min)
1	50	1	203.2 ± 1.5	59.6 ± 3.7
2	100	0.71	141.2 ± 0.6	206.8 ± 2.7
3	50	0.42	134.5 ± 1.0	203 ± 1.3
4	0	0.71	239.3 ± 0.8	57.2 ± 3.4
5	85.4	0.92	183.8 ± 0.2	105.9 ± 2.0
6	85.4	0.5	86.6 ± 1.0	251.4 ± 3.0
7	14.6	0.5	224.9 ± 0.2	102.3 ± 1.6
8	14.6	0.92	226.2 ± 0.5	53.3 ± 7.1
9	50	0.71	206.4 ± 1.0	100.9 ± 2.2
10	50	0.71	206.6 ± 0.7	90.7 ± 2.5
11	50	0.71	206.4 ± 1.0	92.2 ± 2.7

**Table 3:** ANOVA table for the surface response quadratic model (response: T_g_).

Source	Sum of Squares	df	Mean Square	F Value	*p*-Value (Prob > F)
Model	21,837.58	5	4367.52	97.95	<0.0001 (Significant)
A-BPF in BPF/TGPAP	2730.24	1	2730.24	61.23	0.0005
B-Stoichiometry	580.89	1	580.89	13.03	0.0154
AB	2296.01	1	2296.01	51.49	0.0008
A^2^	353.05	1	353.05	7.92	0.0374
B^2^	1960.37	1	1960.37	43.96	0.0012
Residual	222.92	5	44.59		
Std. Dev.	6.68	R^2^	0.9899		
Mean	187.19	Adj. R^2^	0.9798		
C. V. %	3.57	Pred R^2^	0.9281		
PRESS	1585.26	Adeq. Precision	31.541		

**Table 4:** ANOVA table for the surface response quadratic model (response: PW).

Source	Sum of Squares	df	Mean Square	F Value	*p*-Value (Prob > F)
Model	46,138.06	5	9227.61	441.82	<0.0001 (Significant)
A-BPF in BPF/TGPAP	2811.65	1	2811.65	134.62	<0.0001
B-Stoichiometry	6331.62	1	6331.62	303.16	<0.0001
AB	2327.10	1	2327.10	111.42	0.0001
A^2^	1801.80	1	1801.80	86.27	0.0002
B^2^	1727.93	1	1727.93	82.73	0.0003
Residual	104.43	5	20.89		
Std. Dev.	4.57	R^2^	0.9977		
Mean	120.29	Adj. R^2^	0.9955		
C. V. %	3.80	Pred R^2^	0.9903		
PRESS	449.79	Adeq. Precision	60.041		

**Table 5:** Criteria for epoxy blend optimization (a greater number of * indicate that a higher importance was placed on that particular response or factor).

Factor/Response	Goal	Lower Limit	Upper Limit	Importance
BPF in BPF/TGPAP	Maximize	0	100	****
Stoichiometry	Minimize	0.42	1	***
T_g_ infinity	Is in range	180	185	*****
Processing window	Maximize	53.28	251.4	*****

## Data Availability

Data are contained within the article.
